# HIV transfer between CD4 T cells does not require LFA-1 binding to ICAM-1 and is governed by the interaction of HIV envelope glycoprotein with CD4

**DOI:** 10.1186/1742-4690-5-32

**Published:** 2008-03-31

**Authors:** Isabel Puigdomènech, Marta Massanella, Nuria Izquierdo-Useros, Raul Ruiz-Hernandez, Marta Curriu, Margarita Bofill, Javier Martinez-Picado, Manel Juan, Bonaventura Clotet, Julià Blanco

**Affiliations:** 1 Fundació irsiCaixa, Institut de Recerca en Ciències de la Salut Germans Trias i Pujol (IGTP), Hospital Germans Trias i Pujol, Universitat Autònoma de Barcelona, Badalona 08916, Barcelona, Catalonia, Spain; 2 ICREA, Fundació irsiCaixa, Hospital Germans Trias i Pujol, Badalona 08916 Barcelona, Catalonia, Spain; 3 LIRAD, Institut de Recerca en Ciències de la Salut Germans Trias i Pujol (IGTP), Hospital Germans Trias i Pujol, Universitat Autònoma de Barcelona, Badalona 08916 Barcelona, Catalonia, Spain; 4 Servei d'Immunologia. CDB – Hospital Clínic, Villaroel 170, 08036 Barcelona, Spain

## Abstract

**Background:**

Cell-to-cell HIV transmission requires cellular contacts that may be in part mediated by the integrin leukocyte function antigen (LFA)-1 and its ligands intercellular adhesion molecule (ICAM)-1, -2 and -3. The role of these molecules in free virus infection of CD4 T cells or in transinfection mediated by dendritic cells (DC) has been previously described. Here, we evaluate their role in viral transmission between different HIV producing cells and primary CD4 T cells.

**Results:**

The formation of cellular conjugates and subsequent HIV transmission between productively infected MOLT cell lines and primary CD4 T cells was not inhibited by a panel of blocking antibodies against ICAM-1, ICAM-3 and α and β chains of LFA-1. Complete abrogation of HIV transmission and formation of cellular conjugates was only observed when gp120/CD4 interactions were blocked. The dispensable role of LFA-1 in HIV transmission was confirmed using non-lymphoid 293T cells, lacking the expression of adhesion molecules, as HIV producing cells. Moreover, HIV transmission between infected and uninfected primary CD4 T cells was abrogated by inhibitors of gp120 binding to CD4 but was not inhibited by blocking LFA-1 binding to ICAM-1 or ICAM-3. Rather, LFA-1 and ICAM-3 mAbs enhanced HIV transfer. All HIV producing cells (including 293T cells) transferred HIV particles more efficiently to memory than to naive CD4 T cells.

**Conclusion:**

In contrast to other mechanisms of viral spread, HIV transmission between infected and uninfected T cells efficiently occurs in the absence of adhesion molecules. Thus, gp120/CD4 interactions are the main driving force of the formation of cellular contacts between infected and uninfected CD4 T cells whereby HIV transmission occurs.

## Background

Cell-to-cell HIV transmission is a major determinant of HIV spread *in vivo *[1] and is required for efficient HIV replication *in vitro *[2]. Although free HIV particles are infectious, they show a short lifespan at 37°C [3] and lower infectivity than cell-to-cell HIV transmission [4]. Cell-to-cell virus transmission occurs through the formation of stable cellular contacts defined as virological synapses [5], that can be formed between a target CD4 T cell and either a dendritic cell (DC) or a productively HIV infected cell. Although both synapses share the common function of transmitting HIV to CD4 T cells, their structures appreciably differ: DC-T cell synapses concentrate TCR/MHC complexes in the central supramolecular activation cluster (cSMAC), while in T cell-T cell synapses the cSMAC is formed by the binding of HIV envelope glycoprotein (Env) to CD4 [5,6].

LFA-1 appears to play a key role in the formation of virological synapses by interacting with its high-affinity ligand CD54/ICAM-1 [7-9]. The binding of ICAM-1 to LFA-1 is facilitated by initial low-affinity interactions of LFA-1 with the widely expressed ligand CD50/ICAM-3 at the cSMAC [10,11] that leads to LFA-1 activation and clustering in the periphery of the synaptic structures (peripheral supramolecular activation cluster or pSMAC) stabilizing cellular contacts and providing costimulatory signals [8,12]. However, recent work suggests that the cSMAC of immunological synapses may be built in the absence of LFA-1 [12].

The active contribution of LFA-1/ICAM-1 interaction to HIV spread has been described during free virus infection of CD4 T cells and infection mediated by DC. In both cases, LFA-1 increases viral infectivity [9,13] and directs infection towards CD45RO^+ ^memory CD4 T cells [14,15]. However, the involvement of adhesion molecules in the transmission of HIV between infected and uninfected CD4 T cells is poorly defined: although LFA-1 may modulate the formation of cellular conjugates and synaptic structures, a clear correlation between LFA-1 expression and HIV transmission has not been described [8].

Cellular contacts between infected and uninfected primary CD4 T cells lead to the polarization of cell-surface Env expression and viral budding [5,6,16] towards the contact area. Concomitant polarization of CD4 results in the formation of a virological synapse [17]. This synaptic structure allows high levels of viral transfer between infected and target cells [18,19] which, aside from increasing viral entry, also activates endocytic mechanisms of HIV capture that require the extracellular but no the intracellular moiety of CD4 [18].

To define the contribution of adhesion molecules to the process of HIV transfer between infected and uninfected CD4 T cells, we have cultured primary CD4 T cells with different productively infected cell lines or primary cells. Our data suggest that, in contrast to other mechanisms of HIV spread, the interaction of LFA-1 with its main ligand ICAM-1 is dispensable for HIV transmission between CD4 T cells.

## Results

### The role of adhesion molecules in cell-to-cell contacts between HIV infected and uninfected T cells

**Figure 1:**
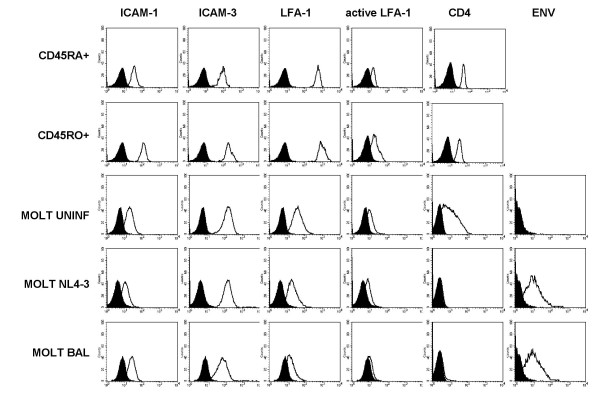
**The expression of adhesion molecules in effector and targets cells**. Primary CD4 T cells as well as MOLT cells uninfected or chronically infected with NL4-3 or BaL isolates were analyzed for the expression of ICAM-1, ICAM-3, total LFA-1, the activated form of LFA-1 and CD4. Staining of the cell surface molecules was performed using monoclonal antibodies RM3A5, 140.11, R7.1, mAb24 and Leu3a respectively. The expression of HIV Env was evaluated in MOLT cells using pooled serums from HIV infected individuals. Figure shows the expression of each individual antigen (empty peaks) with the negative control of staining (solid peaks). Histograms show a single representative experiment.

The known role of LFA-1 in HIV attachment [13] and formation of synaptic structures [5] led us to evaluate the role of adhesion molecules in our previously described model of T cell-to-T cell HIV transmission [18]. First, we confirmed the expression of LFA-1 (total and activated forms), ICAM-1 and -3 in target purified primary CD4 T cells and effector MOLT cells. All cells stained positive for these antigens with different intensities. Primary CD4 T cells expressed high levels of LFA-1 and lower levels of ICAM-3, ICAM-1 and activated LFA-1. Memory cells showed higher expression of all antigens compared to naive cells (Figure 1). All MOLT cells (uninfected and infected with either NL4-3 or BaL viruses) expressed lower levels of LFA-1 (total and activated forms) than primary CD4 T cells, while ICAM-1 and ICAM-3 expression was comparable to that of naive CD4 T cells (Figure 1). The expression of CD4 and HIV Env was also evaluated. Cell surface CD4 staining served to confirm similar levels of expression in naive and memory primary cells, and the complete downregulation in productively infected cells (Figure 1). Env expression was comparable in both NL4-3 and BaL infected MOLT cells.

**Figure 2:**
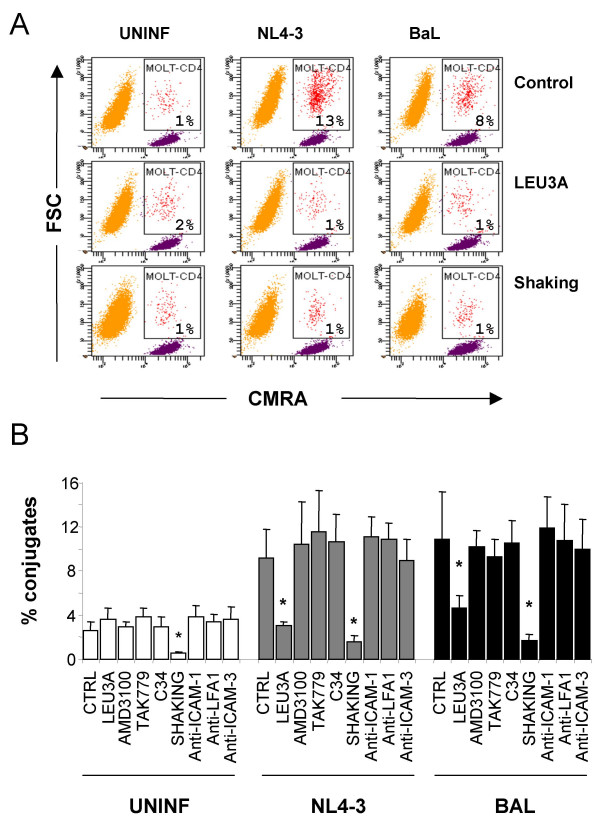
**Formation of T cell-T cell conjugates**. Panel A shows a representative experiment of quantification of cellular contacts between MOLT cells and purified CMRA-labeled CD4 T cells. MOLT cells appear as an unstained population with high forward scatter values (in orange); while CD4 T cells were identified by fluorescent staining and low forward scatter values (in purple). Events displaying bright fluorescence and forward scatter values consistent with MOLT cells were defined as cellular conjugates (in red, gate MOLT-CD4 in the figure). The percentage of CD4 T cells forming conjugates in the absence or the presence of Leu3a, or under continuous shacking condition is shown in each plot. Panel B shows the quantification of cellular conjugates in the presence of the following inhibitors: mAbs against the adhesion molecules ICAM-1 (RM3A5), ICAM-3 (140.11) and LFA-1 (R7.1), coreceptor antagonists AMD3100 and TAK779 (all at 10 μg/ml), gp41 inhibitor C34 (1 μg/ml) or Leu3a (5 μg/ml). Data are Mean ± SD of 3 independent experiments including cells from 3 different donors. Asterisks indicate a significant inhibition in the formation of conjugates in the presence of Leu3a and under shaking conditions.

Since the potential effect of adhesion molecules on HIV transmission is expected to be associated with the initial steps of cellular contacts, we have developed a flow cytometry method to quantify cellular conjugates formed between MOLT and fluorescently-labeled CD4T cells. In these experiments, individual cells could be identified by forward scatter and fluorescence values. Cellular conjugates displayed high levels of fluorescence (given by the labeled CD4 T cells) and forward scatter values similar to the MOLT cell population (Figure 2A). We employed uninfected MOLT cells as control and also analyzed the effects of different inhibitors. MOLT NL4-3 and BaL cells showed higher percentage of cellular conjugates (13% and 8% respectively) than uninfected cells (1%) (Figure 2A, Control). Regardless of viral tropism, cellular conjugates were significantly (p < 0.05) inhibited by the anti-CD4 mAb Leu3a or by continuous shacking during incubation as described by Sourisseau et al [2] (Fig 2A). In contrast, the addition of antagonists of either CXCR4 (AMD3100) or CCR5 (TAK779) and the gp41 inhibitor C34 (at concentrations that completely blocked cell-to-cell fusion) failed to inhibit the formation of cellular conjugates (Figure 2B). Similarly, the addition of blocking antibodies against adhesion molecules (LFA-1, ICAM-1 and -3), did not show any significant impact on the formation of cellular conjugates. As a whole, these data suggest that the expression of HIV Env and CD4 are the main determinants of the formation of cellular conjugates between infected and uninfected CD4 T cells.

### The role of adhesion molecules in HIV transfer between CD4 T cells

**Figure 3:**
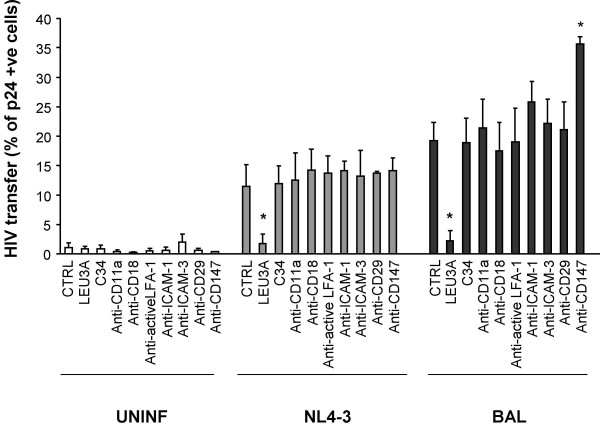
**The role of adhesion molecules on T cell-to-T cell HIV transmission**. Blocking antibodies against the indicated cell surface molecules were used to inhibit the transfer of HIV particles after 2 h of coculture of primary CD4 T cells with uninfected MOLT (white bars), MOLT NL4-3 (grey bars) or MOLT BaL cells (dark bars). Concentrations were 10 μg/mL for all mAbs against cell surface molecules, except for Leu3a (5 μg/mL) and C34 (1 μg/mL). Data are Mean ± SD of 3 independent experiments employing cells of 3 different donors. Asterisks indicate a significant inhibition in HIV transmission in the presence of Leu3a.

Recently, Jolly et al [8] have suggested that during HIV transmission between CD4 T cells, adhesion molecules may modulate not only the formation of cellular conjugates but also the function of virological synapses [8]. To address this possibility in our culture system, we tested the effect of antibodies blocking adhesion molecules in an assay that measures transfer of HIV particles from infected to uninfected T cells. The blockade of the α or β chains of LFA-1 (CD11a and CD18, respectively), the activated form of LFA-1, and ICAM-1 or ICAM-3 did not show any inhibitory effect on the transfer of HIV particles, measured as the percentage of p24 positive target cells (Figure 3). Similarly, combinations of anti-ICAM-1 with either anti-ICAM-3 or anti-LFA-1 antibodies failed to block HIV transfer (data not shown). In the search of other adhesion components expressed on the cell surface, which could be involved in the formation of cellular conjugates or HIV attachment, we have also evaluated the role of CD147 and CD29 [20,21]. Blocking antibodies against these molecules did not inhibit HIV transfer; rather a significant increase was observed in the presence of the anti-CD147 antibody (Figure 3).

**Figure 4:**
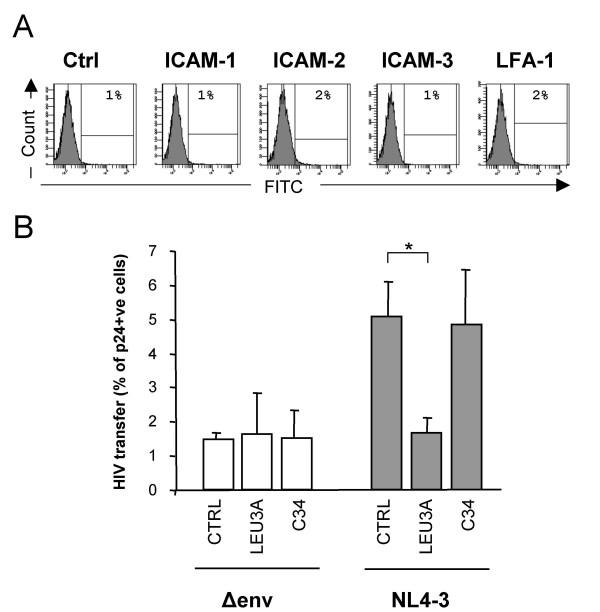
**LFA-1 independent cell-to-cell HIV transmission**. Panel A depicts the expression of the indicated adhesion molecules on the surface of the 293T cell line. The percentage of positive cells is indicated in each histogram. Control histogram corresponds to background of staining. Panel B shows HIV transfer from 293T cells, transfected to produce HIV particles, to target CD4 T cells in the absence (light bars) or presence (grey bars) of NL4-3 Env expression. The effect on the addition of blocking antibodies against CD4 (Leu3a) and the gp41 inhibitory peptide C34 was also tested. Data (Mean ± SD) were obtained using cells from 3 different donors. Asterisk indicates a significant inhibition in HIV transmission in the presence of Leu3a within NL4-3 Env transfected 293T cells.

The failure of antibodies against adhesion molecules to inhibit the formation of cellular conjugates and the blocking effect of Leu3a suggested that gp120 binding to CD4 is the main driving force of the formation of T cell-to-T cell virological synapses. Therefore, T cell-to-T cell HIV transmission might take place in the absence of LFA-1 interactions with ICAMs. To investigate the requirements of LFA-1 function in HIV transmission, we used 293T cells. First, we confirmed that these cells do not show detectable expression of LFA-1, ICAM-1, -2 and -3 on their surface (Figure 4A). Second, we transfected 293T cells with plasmids coding for an *env *deficient genome of HIV (Δenv) together with an *env/rev *cassette (NL4-3 isolate). Of note, 293T cells transfected with the plasmid Δenv alone were used as a control of potential Env-independent nonspecific transmission. Transfected cells were then cocultured with primary CD4 T cells to analyze cell-to-cell viral transfer. HIV was specifically transferred from Env-expressing 293T to CD4 T cells by a mechanism similar to that observed for MOLT infected cells: it was abrogated by Leu3a and unaffected by the addition of C34 (Figure 4B), reasserting that the binding of LFA-1 to its ligands is not necessary to observe an efficient HIV transmission.

### Selectivity of LFA-1 independent HIV transfer

**Figure 5:**
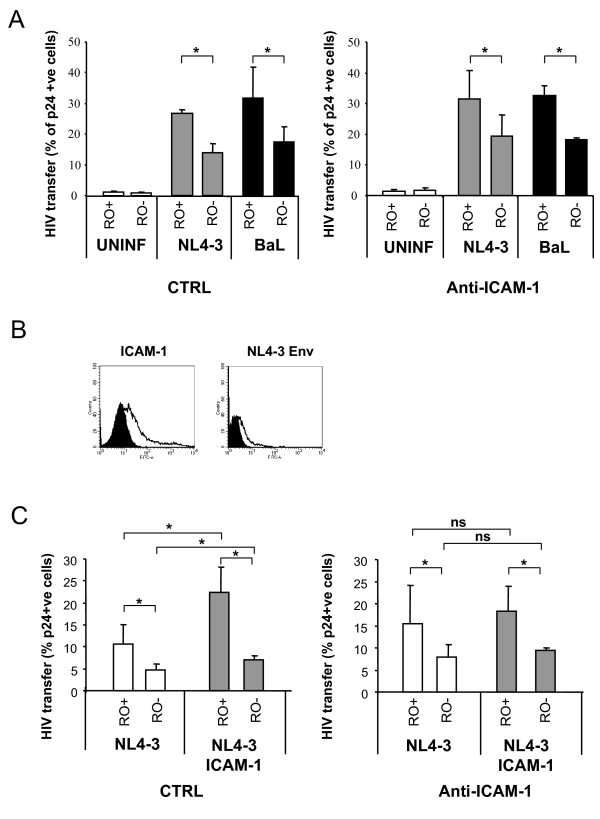
**Preferential LFA-1-independent transfer of HIV particles to CD45RO+ cells during T cell-T cell synapses**. Panel A. MOLT cells chronically infected with NL4-3 (grey bars) and BaL (dark bars) viruses or left uninfected (white bars) were cultured with purified primary CD4 T cells. After 2 h of coculture, cells were stained with anti-CD45RO and anti-HIV p24 antibodies. Analysis of HIV transfer in the absence (left graph) or presence (right graph) of the RM3A5 blocking mAb against ICAM-1 was performed after gating separately CD45RO and CD45RA CD4 T cells. Panel B shows the expression of HIV Env (right) and ICAM-1 (left) in 293T transfected cells (empty peaks), solid peaks correspond to negative controls of staining. Panel C shows HIV transfer from 293T cells transfected with an Env defective and an NL4-3 Env plasmids, to CD45RO- (RO-) and CD45RO+ (RO+) target cells in the absence (light bars) or presence (grey bars) of ICAM-1 expression. HIV transmission was again measured in the absence (left graph) or the presence (right graph) of the blocking RM3A5 antibody against ICAM-1. Values are Mean ± SD of 3 experiments performed with CD4 T cells from 3 different donors. Asterisks denote significant differences in HIV transmission to the CD45RA CD4 T subset compared to CD45RO cells (Panel A and C). Significant differences intrasubsets induced by ICAM-1 expression are also indicated by asterisks in panel C, while ns denotes no statistical significance.

Since the expression of adhesion molecules in memory cells is higher compared to the naïve subset (Figure 1), the active contribution of LFA-1 to the process of HIV spread has been associated to the higher susceptibility of CD4 CD45RO T cells to HIV attachment and infection [14]. Therefore, we sough to evaluate whether LFA-1-independent HIV transfer between CD4 T cells might present a different target cell selectivity. In this set of experiments, after coculture with HIV producing cells, we gated memory (CD45RO+) and naïve (CD45RO-) cells and then we calculated the percentage of p24 positive cells in each subset. Figure 5A (left) shows that memory CD4 T cells had significantly higher levels of p24 than naive cells when cocultured with both NL4-3 and BaL chronically infected MOLT cells (p < 0.05). This selectivity of HIV transfer towards the memory subset was independent of LFA-1 binding to ICAM-1, as it was not modified by the addition of the anti-ICAM-1 mAb RM3A5 (Fig 5A, right).

Since 293T cells have been used as a standard model to evaluate the role of ICAM-1 in HIV infection [13,14], we cotransfected these cells with a plasmid coding for ICAM-1. The expression of HIV Env and ICAM-1 was assessed by flow cytometry, being ICAM-1 expressed with more efficiency (26 ± 6% ICAM-1 expressing cells vs. 14 ± 3% Env expressing cells, with relative fluorescence intensity (RFI) values of 4.3 ± 2.3 and 2.1 ± 0.4, respectively, Figure 5B). The selective transfer to memory cells was still observed in the absence of adhesion molecules by using 293T cells transfected as in Figure 4. Once more, memory T cells showed significant higher values of p24 content than naïve cells (Figure 5C, left). High levels of ICAM-1 expression in effector cells significantly increased the efficiency of HIV transmission to both naïve (1.2 fold) and memory T cells (2.1 fold) (Figure 5C, left). HIV transfer mediated by ICAM-1 expressing 293T cells was not modified by C34, and was inhibited by Leu3a (data not shown). Consistent with a specific role of ICAM-1 in this cellular model, the anti-ICAM-1 mAb RM3A5 reverted the net increase of HIV transfer and the higher selectivity towards memory cells induced by ICAM-1 expression. However, this antibody was unable to completely block HIV transfer (Figure 5C, right). These data support the idea that the role of ICAM-1 is cell type dependent and provide a positive control for the inhibitory effect of the anti-ICAM-1 mAb RM3A5.

### HIV transmission between primary CD4 T cells

**Figure 6:**
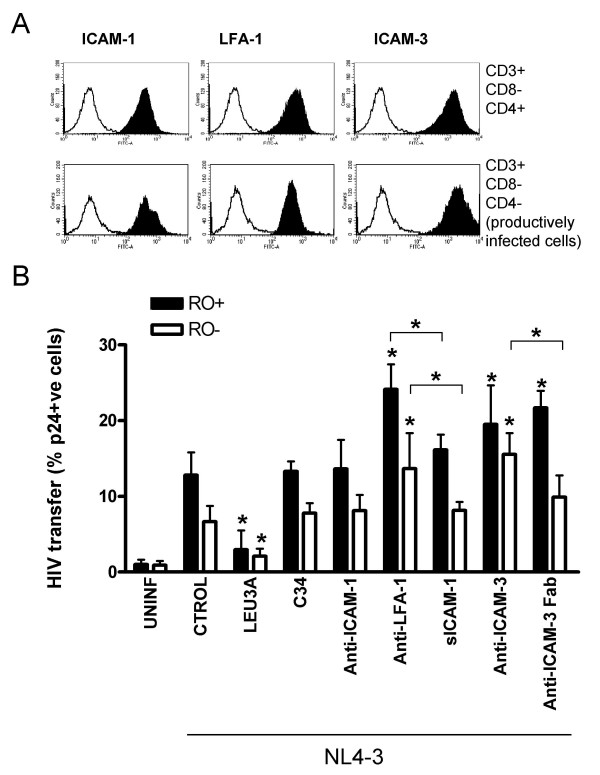
**The role of adhesion molecules in HIV transmission between primary CD4 T cells**. In panel A the expression of the indicated adhesion molecules was analyzed in the surface of T cells from a representative NL4-3 infected PBMC culture. Cells were gated as CD3+/CD8-/CD4+ PBMCs (non-productively infected cells, upper histograms) or CD3+/CD8-/CD4- PBMCs (productively infected cells, lower histograms). Histograms show a representative experiment displaying the expression of each individual antigen (solid peaks) with the negative control of staining (empty peaks). In panel B, purified productively HIV infected CD3+/CD8-/CD4- PBMCs were cocultured with CMFDA-labeled primary unstimulated CD4 T cells. After 24 hours of coculture cells were stained with anti-CD45RO and anti-HIV CA p24 antibodies. HIV transmission was measured in both memory CD45RO+ (RO+ solid bars) and naive (RO-, empty bars) subsets in the presence of Leu3a, C34 and a panel of blocking agents against adhesion molecules used at the same concentrations as in Figure 3 (whole IgGs against ICAM-1, LFA-1 and ICAM-3, soluble ICAM-1 or Fab fragments of the anti-ICAM-3 mAb 140.11). Values are Mean ± SD of data corresponding to up to 6 different donors. Asterisks indicate significant differences (p < 0.05) from control or between divalent and monomeric blocking agents.

The different role of ICAM-1 observed in HIV transfer from MOLT or from 293T cells to CD4 T cells could be due to a different balance between the expression of ICAM-1 and HIV Env on the surface of these cells. In fact, 293T cells expressed lower levels of Env than ICAM-1 both in terms of percentage of positive cells and RFI (Figure 5B). In contrast, the expression of ICAM-1 is low in MOLT cells, which in turn express higher levels of HIV Env (RFI values of 2.65 and 6.95 for ICAM-1 and Env, respectively in MOLT NL4-3 infected cells, Figure 1). To clearly define the role of ICAM-1 in HIV transmission between primary CD4 T cells, we purified productively infected CD4 T cells from infected cultures of peripheral blood mononuclear cells (PBMC) and used them as effector cells. First, we determined the expression of LFA-1 (mAb R7.1), ICAM-1 (mAb RM3A5) and ICAM-3 (mAb 140.11) in gated CD3+CD8-CD4- T cells of infected PBMC cultures, which represent the productively infected cell population [18]. The expression of these molecules was compared to the CD3+CD8-CD4+ cells of the same cultures, finding no significant differences (Figure 6A). Interestingly, the expression of ICAM-1 and LFA-1 was higher than in MOLT cells (Figure 1). Next, we purified productively infected CD3+/CD8-/CD4- T cells that were cocultured with unstimulated uninfected CD4 T cells. Transfer of HIV was again detected in both naïve and memory subsets, being significantly (p < 0.05) higher in the latter subset and significantly (p < 0.05) inhibited by Leu3A but not C34 (Figure 6B). Similarly to MOLT cells, the addition of blocking ICAM-1, LFA-1 or ICAM-3 antibodies did not inhibit HIV transfer; rather significant increases were induced by the 140.11 anti-ICAM-3 or the R7.1 anti-LFA-1 antibodies (Figure 6B). To address the role of antibody mediated cross linking and signaling on these enhancing effects, we compared the effect of Fab fragments of the ICAM-3 mAb 140.11 or a monomeric soluble form of ICAM-1 with the effect of whole IgGs. The increase in HIV transfer induced by anti-LFA-1 R7.1 IgG was significantly lost when LFA-1 was blocked using sICAM-1 (Figure 6B). Similarly, significant differences were observed between the enhancing effect of the anti-ICAM-3 140.11 IgG and its Fab fragment, although the latter still retained some ability to increase HIV transfer (Figure 6B). None of the inhibitors used, unless the anti-CD4 mAb Leu3a, inhibited HIV transfer. In summary, these data support the idea that the binding of HIV Env (gp120) to CD4 governs HIV transfer between CD4 T cells in the absence of LFA-1 interactions with ICAM-1 or -3. Nevertheless, signaling through these adhesion molecules may modulate both the extent and the target cell selectivity of viral transfer.

## Discussion

We provide several lines of evidence that highlight the relevance of gp120 binding to CD4 as a primary determinant in the formation of T cell-T cell conjugates over the role of HIV coreceptors CXCR4 and CCR5 or the adhesion molecules studied here. Concerning coreceptors, it is well known that CXCR4 is highly expressed in cultured primary T cells, while CCR5 is restricted to a subset of memory cells [22]. Therefore, a potential active participation of the coreceptor in cellular conjugates should favor X4 Envs. However, we show that both X4 and R5 Envs formed similar amounts of cellular conjugates with primary CD4 T cells (Figure 2). Moreover, coreceptor blockade did not inhibit cellular contacts, which were only sensitive to CD4 blockade. Thus, the implication of coreceptors in HIV transmission is subsequent to the cellular contact and determine further cell-to-cell fusion, productive infection or death of target cells [23].

The potential role of adhesion molecules (mainly LFA-1 and ICAM-1) is unexpectedly secondary for HIV transfer from MOLT cells or primary infected T cells to CD4 T cells. We have observed that Env-independent contacts between uninfected MOLT cells and CD4 T cells are near background levels (Figure 2) and that the blockade of adhesion molecules, employing inhibitory specific mAbs, did not significantly modify HIV Env-mediated cellular contacts or HIV transfer (Figure 2, 3). Noteworthy, all antibodies were titrated and added at saturating concentrations in inhibitory experiments. As a positive control, activity was assessed in parallel experiments in which LFA-1 and ICAM-1 antibodies efficiently blocked monocyte aggregation induced by IL-12 and IL-18 (data not shown, G Coma, unpublished results) [24]. Moreover, the anti-ICAM-1 mAb RM3A5 inhibited the modest effect (2-fold increase in HIV transfer to memory cells) observed when ICAM-1 is expressed in 293 T cells (Figure 5C).

The secondary role of ICAM-1 in HIV transfer between CD4 T cells and its limited effect in 293T cells is contradictory with the more relevant role reported in the infection of CD4 T cells by free HIV particles [13], and the complete requirement of this molecule for transinfection induced by DC [9]. Of note, T cell-T cell contacts and DC-T cell contacts are relatively different processes. We have also worked with DC, and we have found that, in contrast to T cell-T cell contacts, high level of DC-T cell contacts are observed in the absence of HIV Env (IP, NIU unpublished results) [9]. In fact, for DC mediated transinfection of CD4 T cells, HIV takes advantage of naturally occurring cellular contacts during DC scanning of T cells or antigen presentation. In contrast, for efficient transmission between T cells, HIV forces the contacts through Env expression on the surface of infected cells, as T cell-T cell contacts are low in the absence of HIV Env. In this regard, Env expression has been recently reported to increase the number of effective contacts between infected DC and T cells [25] despite the relevant role of adhesion molecules in this type of synapses [26,27]. It is therefore reasonable that Env plays a key role in the formation of conjugates between CD4 T cells that *in vitro *have shown low levels of Env-independent contacts. *In vivo*, adhesion molecules may also actively participate in antigen-dependent mechanisms of HIV spread between CD4 T cells, involving regulatory mechanisms or antigen presentation. However, the contribution of T cell contacts to regulatory mechanisms is unclear [28], and antigen presentation is impaired by Nef-induced down regulation of MHC Class II molecules and the fast kinetics of Env expression in infected cells [29,30].

The contribution of adhesion molecules to HIV spread has been widely studied. Early reports suggest a role in syncytium formation but not in HIV spread or replication [31,32]. Also efficient Env functions, such as cell-to-cell fusion or HIV transfer, have been reported in LFA-1-deficient cells or expressing low affinity forms of this integrin [8,31]. A more recent report focused on the formation of virological synapses suggests that cellular conjugates and cell-to-cell HIV transmission were poorly affected by the blockade of adhesion molecules, and that only morphological determinants of virological synapses appear to be inhibited [8]. Our results fully agree with a secondary role for adhesion molecules in the formation of cellular conjugates and the transfer of HIV particles between infected and uninfected T cells, and demonstrate that the binding of Env to CD4 governs the formation of cellular conjugates. Interestingly, signaling through LFA-1 and ICAM-3 appear to modulate the extent and the selectivity of HIV transfer between primary CD4 T cells, an effect that may be relevant for HIV spread *in vivo*.

Our data point to a different role of ICAM-1 in HIV transfer from MOLT or 293T cells to CD4 T cells. One can speculate that cellular contacts and virus transmission are regulated by a balance between the expression of Env and adhesion molecules on effector cells. When HIV is presented by 293T cells we detected additive contributions of both Env and ICAM-1 to HIV transfer, most likely due to the higher expression of ICAM-1, which may increase the number or cellular contacts or alternatively may signal through LFA-1 in target cells. In contrast, during HIV presentation by infected CD4 T cells (primary or MOLT cells), this equilibrium is completely unbalanced towards a full control of HIV transmission by Env binding to CD4.

By analogy to cell-free HIV attachment, which preferentially targets memory CD4 T cells [14,33], we have also analyzed the selectivity of cell-to-cell HIV transfer for CD4 T cell subsets. Unexpectedly, MOLT, 293T and primary infected cells targeted CD45RO+ CD4 T cells by a mechanism independent of LFA-1 (Figures 5 and 6). A potential explanation could be associated with the organization of signaling molecules, including ZAP70, which is more efficiently recruited to the immunological synapse in memory cells [34]. Interestingly, ZAP70 signaling has been also involved in the formation of virological synapses and cell-to-cell HIV spread [35].

## Conclusion

The transfer of HIV particles between infected and uninfected CD4 T cells is governed by HIV Env binding to CD4 and selectively targets memory cells. Adhesion molecules LFA-1 and ICAM-1 or -3 are not strictly required for HIV transfer but their signaling capacity modulates the extent and selectivity of HIV transfer.

## Materials and Methods

### Cells

Peripheral blood cells from healthy blood donors were provided by the Banc de Sang i de Teixits (BST) and obtained after approval from the Ethical Committee of the Center. Peripheral blood mononuclear cells (PBMC) were obtained from a standard Ficoll density gradient purification. Mononuclear cells were immediately used for further purification of T cell subsets. Whole CD4 T cells, memory CD4 T cells (CD45RO+) and naïve CD4 T cells (CD45RA+) were purified by negative immunomagnetic selection using the appropriate cocktails of antibodies (Miltenyi Biotec SL, Madrid, Spain). Purity of isolated populations (> 95%) was assessed by flow cytometry after CD4, CD45RO and CD45RA staining (BD Biosciences). Primary cells were cultured in RPMI1640 medium supplemented with 10% heat inactivated fetal calf serum (FCS, Invitrogen, Barcelona, Spain) in the absence of any other stimuli.

The T-lymphoblastoid MOLT cell lines chronically infected with X4 or R5-tropic HIV-1 have been previously described [18,23]. Envelopes used were NL4-3 and BaL as prototypic X4 and R5 HIV isolates, respectively. HEK 293T cells (obtained from the NIH AIDS Reagent Program) were cultured in DMEM. All media were supplemented with 10% heat inactivated FCS and selection antibiotics when required.

### Antibodies and determination of cell-surface expression

The expression of the adhesion molecules was studied using the following antibodies: anti-CD11 (LFA-1 α chain, mAb HI111, Ebioscience, San Diego, USA), anti-CD18/LFA-1 β chain mAb 68.5A5 [36] anti-LFA-1 active form mAb24 (Abcam, Oxford UK) [37], anti-CD54/ICAM-1 RM3A5 (kindly provided by Dr. R. Vilella, Hospital Clínic, Barcelona, Spain) [38,39], anti-ICAM-2 mAb CBR-IC2/2 (Axxora, Switzerland), anti-CD50/ICAM-3 140.11 (kindly provided by Dr. R. Vilella, Hospital Clínic, Barcelona, Spain) [40], anti-CD29 mAb P4C10 (Chemicon, Hamphsire UK) and anti-CD147 mAb HIM6 (BD, Madrid Spain). Cells were incubated for 20 min, washed with PBS and incubated with FITC-labeled goat anti-mouse (BD Bioscience, Madrid, Spain). The FITC-labeled goat anti-mouse was used as negative control. Cells were finally washed twice again with PBS and fixed with formaldehyde 1% before flow cytometry analysis. Expression was determined in effector MOLT cells (uninfected or HIV-1-infected), 293T cells and PBMCs (uninfected or HIV-1-infected) and in target CD4 T cells. After titration by flow cytometry, the above-described antibodies were used at saturating concentrations in coculture of effector cells with primary CD4 T cells to evaluate their effect on HIV transfer. Expression of CD4 was determined using the anti-CD4 mAb Leu3a (BD Bioscience, Madrid, Spain). The expression of HIV Env was determined by using a pool of serums from HIV infected patients (dilution 1/10), after washing unbound antibodies, Env expression was revealed using a FICT-coupled goat anti-human antibody (Dako Cytomation, Barcelona, Spain). For quantitative evaluation of cell surface expression, Relative Fluorescence Intensity values were calculated by dividing the Mean Fluorescence Intensity of positive and negative controls of staining.

In some experiments sICAM-1 (R&D Systems, Minneapolis, MN) and Fab fragments of ICAM-3 mAb 140.11 were also used. Fab 140.11 was obtained by digestion of 10 mg of whole IgG using a papain-based commercial kit (Fab Preparation Kit, Thermo Scientific, Barcelona, Spain) following manufacturer's instructions. After digestion, Fab was purified using a Protein A column that yielded 90% pure Fab preparations, as assessed by SDS-PAGE. Main contaminant were Fc fragments, without detectable IgG.

### Measuring cellular conjugates

To analyze the contacts established between uninfected or infected MOLT cells and primary target cells, purified CD4 T cells were first labeled with the cell tracker CMRA (Molecular Probes, Leiden, NL) during 30 minutes, washed twice with PBS and left overnight at 37°C. Then, stained cells were cocultured with MOLT cells (200,000 cells of each type) for 2 hours at 37° with or without shaking conditions on a 96 well flat-bottom plate. Inhibitors tested were: the anti-CD4 mAb Leu3a (BD Biosciences, 1 μg/ml), the gp41 inhibitor C34 (Service of Peptide Synthesis, University of Barcelona, 1 μg/ml), the CXCR4 antagonist AMD3100 and the CCR5 antagonist TAK779 (both obtained through the NIH AIDS Reagent Program and used at 10 μg/ml). The following mAbs R7.1 (LFA1), mab24 (activated LFA-1) RM3A5 (ICAM-1) and 140.11 (ICAM-3) were used at 10 μg/ml. Following the incubation time, 50 μL/well of formaldehyde 5% in PBS were added to the cultures without perturbing cellular conjugates, which were analyzed 15 minutes later in a LSRII flow cytometer equipped with a plate loader (BD Bioscience, Madrid, Spain). All the events with similar morphology to MOLT cells and at the same time being positive for the cell tracker label (CMRA) were considered to be stable cellular conjugates between an effector MOLT cell and a target primary CD4 T cell. Percentage of cellular contacts was calculated as follows: {conjugates/(conjugates +CD4 free cells)}x100.

### HIV transmission measures

HIV transfer from infected to uninfected cells was assessed after coculturing 200,000 target primary CD4 T cells with 200,000 effector infected MOLT/CCR5 cells in a final volume of 200 μl. Uninfected MOLT/CCR5 cells were used as control. All assays were performed in 96-well plates using RPMI1640 medium supplemented with 10% FCS. After 2 or 24 hours of culture at 37°C, cells were washed with PBS containing 1% FCS. Cells were fixed, permeabilized (Fix & Perm, Caltag, Burlingame, CA) and stained with PE-labeled KC57 anti-HIV-p24 antigen (p24) mAb (Coulter, Barcelona, Spain). Labeled cells were washed, resuspended in formaldehyde 1% with PBS and analyzed by flow cytometry as described [18]. In some experiments, cells were surface stained with APC-labeled anti-CD45RO antibodies prior to fixation. HIV transfer was independently analyzed after gating CD45RO+ cells (memory) and CD45RO- cells (naïve). Inhibitors were used at the concentrations described above.

### Transfections

Subconfluent 293T cells seeded in a 6-well plate were co-transfected with 4 μg of HIV-1 NL4-3 expression plasmid lacking a functional Env (pNL4-3.Luc.R-E-; NIH AIDS Reagent Program) together with an Env (gp160) expression plasmid pCDNA3-NL4-3 at a ratio 1:2 using a Calphos transfection system (Clontech), adding or not an ICAM-1 expression vector to the transfection (pCDNA3-ICAM-1).

Twenty-four hours post-transfection, 293T cells were cocultured with CD4 primary T cells for 24 h at a ratio 2:1 in the presence or the absence of the inhibitors described above. The level of p24 antigen expression was assessed as described above and was analyzed in 293T cells and CD4 T cells (identified by forward and side scatter values) to determine the efficiency of transfection and the percentage of transmission, respectively. ICAM-1 expression in 293T cells was evaluated as described above using the RM3A5 mAb.

### Isolation of productively HIV infected primary cells

PBMCs from healthy donors were cultured for 72 hours in the presence of 6 U/mL IL-2 (Roche Diagnostics, Spain) and 4 μg/mL phytohemagglutinin (PHA, Sigma-Aldrich, Spain). PBMCs were infected with an X4 virus (multiplicity of infection = 1) during 4 hours, washed twice with PBS and left with RPMI1640 medium supplemented with 10% heat inactivated fetal calf serum containing IL-2 (10 U/mL). In the following days, the expression of p24 antigen, CD3, CD4, and CD8 was monitored by flow cytometry as described [18]. Only CD3+/CD8-/CD4-/p24+ cells were considered to be productively infected because of the complete disappearance of CD4 from the surface of HIV-infected cells. These cells were purified when its percentage reached 3% or above. A combination of cocktail of antibodies used to purify CD4 and CD8 T cells by negative selection (Miltenyi Biotec SL, Madrid, Spain) was used at a ratio 1:1 in order to obtain the desired eluted fraction (CD4-CD8-). Purity of the isolated population was assessed by flow cytometry after CD4 and p24 staining and compared to the retained fraction (CD4+ CD8+). Enriched infected cells were usually > 50% p24 bright.

In order to measure HIV transfer to uninfected CD4 T cells, target cells were labeled with the CMFDA cell tracker (Molecular Probes, Leiden, NL) as described above for CMRA. Cocultures of 300,000 effector CD4-p24+ PBMCs with 150,000 target CD4 T cells were incubated for 24 hours at 37°C 5% CO_2_, and intracellular p24 was measured and analyzed as described above.

### Statistics

Statistic analysis was performed using two-sided Student's *t *test. P values < 0.05 were considered to indicate statistical significance.

## Competing interests

The author(s) declare that they have no competing interests.

## Authors' contributions

IP performed most of experiments and wrote part of the manuscript, MM, JM-P and NIU worked on quantification of cellular contacts, RR-H and MB contributed to the study of cellular subsets, MC designed and prepared Env expression plasmids, MJ contributed to the study of adhesion molecules, BC and JB designed experiments and wrote the manuscript. All authors read and approved the final manuscript.
